# Genome-wide analysis of CNVs in three populations of Tibetan sheep using whole-genome resequencing

**DOI:** 10.3389/fgene.2022.971464

**Published:** 2022-09-07

**Authors:** Linyong Hu, Liangzhi Zhang, Qi Li, Hongjin Liu, Tianwei Xu, Na Zhao, Xueping Han, Shixiao Xu, Xinquan Zhao, Cunfang Zhang

**Affiliations:** ^1^ Key Laboratory of Adaptation and Evolution of Plateau Biota, Northwest Institute of Plateau Biology, Chinese Academy of Sciences, Xining, China; ^2^ Technology Extension Service of Animal Husbandry of Qinghai, Xining, China; ^3^ State Key Laboratory of Plateau Ecology and Agriculture, Qinghai University, Xining, China

**Keywords:** copy number variations, Tibetan sheep, whole genome resequencing, adaptation, domestication

## Abstract

Copy number variation (CNV), an important source of genomic structural variation, can disturb genetic structure, dosage, regulation and expression, and is associated with phenotypic diversity and adaptation to local environments in mammals. In the present study, 24 resequencing datasets were used to characterize CNVs in three ecotypic populations of Tibetan sheep and assess CNVs related to domestication and adaptation in Qinghai-Tibetan Plateau. A total of 87,832 CNV events accounting for 0.3% of the sheep genome were detected. After merging the overlapping CNVs, 2777 CNV regions (CNVRs) were obtained, among which 1098 CNVRs were shared by the three populations. The average length of these CNVRs was more than 3 kb, and duplication events were more frequent than deletions. Functional analysis showed that the shared CNVRs were significantly enriched in 56 GO terms and 18 KEGG pathways that were mainly concerned with ABC transporters, olfactory transduction and oxygen transport. Moreover, 188 CNVRs overlapped with 97 quantitative trait loci (QTLs), such as growth and carcass QTLs, immunoglobulin QTLs, milk yield QTLs and fecal egg counts QTLs. *PCDH15*, *APP* and *GRID2* overlapped with body weight QTLs. Furthermore, Vst analysis showed that *RUNX1*, LOC101104348, LOC105604082 and *PAG11* were highly divergent between Highland-type Tibetan Sheep (HTS) and Valley-type Tibetan sheep (VTS), and *RUNX1* and LOC101111988 were significantly differentiated between VTS and Oura-type Tibetan sheep (OTS). The duplication of *RUNX1* may facilitate the hypoxia adaptation of OTS and HTS in Qinghai-Tibetan Plateau, which deserves further research in detail. In conclusion, for the first time, we represented the genome-wide distribution characteristics of CNVs in Tibetan sheep by resequencing, and provided a valuable genetic variation resource, which will facilitate the elucidation of the genetic basis underlying the distinct phenotypic traits and local adaptation of Tibetan sheep.

## 1 Introduction

Copy number variations (CNVs) and single nucleotide polymorphisms (SNPs), as significant genetic variations, play important roles in domestication and adaptation of animals and plants (Lye and Purugganan, 2019; Merot et al., 2020). Unlike SNPs, which refer to the substitution, deletion or insertion of just a single nucleotide for another, CNVs involve fragmental variation even larger than 1000 bp in size (Iafrate et al., 2004; Feuk et al., 2006). Therefore, it has been widely recognized that CNVs have the potential to markedly affect phenotypic traits of domestic animals (Salehian-Dehkordi et al., 2021).

In the past twenty years, a large number of CNVs have been revealed and identified by using array comparative genome hybridization (aCGH) chips and high-density SNP chips in domestic animals, such as cattle, sheep, goat, pig, horse, dog, chicken, turkey and duck (Bickhart and Liu, 2014; Strillacci et al., 2021). Meanwhile, numerous CNV-overlapping genes have been shown to be associated with coat color (Norris and Whan, 2008), growth, fertility and production (Zhou et al., 2016a; de Lemos et al., 2018a), immune response (Sasaki et al., 2016; Zhang et al., 2016; Chen J. et al., 2017; Chen L. et al., 2017; Xu et al., 2017), olfactory transduction (Zhou et al., 2016b; Upadhyay et al., 2017; de Lemos et al., 2018b; Mielczarek et al., 2018), molecular function (Letaief et al., 2017; Pierce et al., 2018), lipid metabolism (Gao et al., 2017; Xu et al., 2017; de Lemos et al., 2018b; Goyache et al., 2021) and environmental adaptability (Wang et al., 2016). Compared with aCGH and SNP chips, the sensitivity of which is mainly limited by the density of the probes, whole genome resequencing can be used to detect new and rare CNVs (Jenkins et al., 2016). Therefore, an increasing number of CNVs have been detected by using high-throughput sequencing. In terms of sheep, in addition to whole genome detection of new CNVs, the primary focus was uncovering the CNVs associated with economic traits. Yuan et al. found that 1855 CNVRs were associated with 166 quantitative trait loci (QTLs), including milk QTLs, carcass QTLs, and health-related QTLs in fine-wool sheep (Yuan et al., 2021). CNVR overlapping genes, such as *SHE*, *PIGY*, and *BAG4*, were reported to be correlated with body size in sheep (Jiang et al., 2019; Feng et al., 2020; Yang et al., 2021). The CNV-overlapping genes of *BTG3*, *PTGS1* and *PSPH* were involved in fetal muscle development, prostaglandin (PG) synthesis, and bone color (Yang et al., 2018). Meanwhile, the correlation between CNVR-harboring genes and growth traits or phenotypic traits was also attractive. The agouti signaling protein (*ASIP*) gene duplication has been linked to typical white coat color (Norris and Whan, 2008). Distal-less homeobox 3 (*DLX3*) CNV is related to wool curling in Tan sheep (Ma et al., 2017).

Tibetan sheep (TS) is one of the three ancient coarse-wool sheep breeds in China and is predominantly distributed in the Qinghai-Tibetan Plateau (QTP). Even in nowadays, Tibetan sheep, together with yak, provide the Tibetan herders with the main food, fuel and clothing materials. Natural adaptation and artificial selection shaped the TS with the characteristics of adaptation to cold, food shortage, and hypoxia. TS can be divided into three ecotypic populations, namely, Highland-type Tibetan sheep (HTS), Valley-type Tibetan sheep (VTS) and Oura-type Tibetan sheep (OTS) (The photos of the three sheep population were shown in Supplementary Table S1). HTS is famous for its carpet wool production, with an average staple length of up to 25 cm. The proportion of dry dead wool in the OTS is relatively high but has remarkable meat performance. The appearance of VTS is similar to that of HTS but with a smaller body size and shorter staple length. Meanwhile, the VTS was mainly distributed in valley regions with relatively low altitudes (∼2000 m), in contrast to the HTS and OTS (both distributed above 3200 m).

It’s meaningful to elucidate the genomic distribution characteristics and potential functions of CNVs in TS. Nevertheless, there have been few reports on the analysis of CNVs in TS (Huang et al., 2021; Salehian-Dehkordi et al., 2021). Therefore, in the present study, we exploited a large number of CNVs in 24 individuals from the three TS populations using whole genome resequencing and identified CNVs associated with the molecular basis of phenotype differences, domestication and adaptation to QTPs.

## 2 Materials and methods

### 2.1 Animal sampling and genomic DNA sequencing

Twenty-four whole blood samples of Tibetan sheep were collected from their core distribution region in this study, including 8 highland-type sheep (HTS, Qilian County, 4 male and 4 female sheep, similar to the following two sheep groups), 8 valley-type sheep (VTS, Huangyuan County) and 8 outer-type sheep (OTS, Henan County). The samples of each population were randomly collected from more than three different groups and the information was seeked from the herdman to avoid the potential genetic relationship. The collected samples were stored in EDTA antifreezing tubes at -20°C. Genomic DNA was extracted with a QIAamp DNA Blood Mini Kits (Qiagen, Germany), and then the DNA integrity and concentration were checked with 1% agarose gel electrophoresis and Qubit® 3.0 Flurometer (Invitrogen, United States), respectively. A sequencing library was built with 0.2 μg genomic DNA from each sample using the NEB Next® UltraTM DNA Library Prep Kit (NEB, United States) following the manufacturer’s recommendations. Briefly, genomic DNA samples were fragmented by sonication to a size of ∼350 bp, and then DNA fragments were endpolished, A-tailed, and ligated with the full-length adapter, followed by further PCR amplification. The DNA libraries were sequenced using an Illumina HiSeq X-Ten platform (Illumina, United States), and 150 bp paired-end reads were generated and stored in FASTQ format. Paired reads with more than 10% unidentified nucleotides in either read, with low-quality bases (Phred quality value < 5) over 50%, and with more than 10 bp aligned to the adapter were removed by using Fastp (0.19.7) to obtain clean data.

### 2.2 CNV and CNVR detection and annotation

The following steps were required before CNV detection: 1) the clean reads of each sample were aligned against the reference genome of Ovis aries (Oar_v4.0) using BWA (Burrows–Wheeler Aligner) (Li and Durbin, 2009) and 2) alignment files were converted to BAM files using SAMtools software (Li et al., 2009). 3) SAMtools was also used to remove potential PCR duplications. If multiple read pairs have identical external coordinates, only the pair with the highest mapping quality is retained. CNVs were detected using CNVcaller with default parameters (-w: 800 bp, -l: 0.2, -u: 0.7, -g: 0.5) (Abyzov et al., 2011). The individual candidate CNV windows are defined using 2 criteria: 1) The normalized read depth (RD) must be significantly higher or lower than the normalized mean RD (deletions < 1–2 * STDEV; duplications > 1 + 2 * STDEV). 2) Considering that the normalized RD of heterozygous deletions and duplications should be approximately 0.5 and 1.5, respectively, an empirical standard for the normalized RD (deletions < 0.65; duplications > 1.35) must also be achieved. Low-frequency windows with low sequencing coverage were removed, and windows with an allele frequency ≥0.05 or at least 3 homozygous duplicated/deleted individuals were selected for further validation. If Pearson’s correlation index was significant at the *p* = 0.05 level by Student’s t test, these 2 adjacent non-overlapping windows were merged into 1 call. To avoid putative positive CNVRs, the population-level candidate CNVRs for each of the three populations and for TS as a whole was analyzed, respectively. CNV region definition, the distance between the 2 initial calls is less than 20% of their combined length, and the Pearson’s correlation index of the 2 CNVRs is significant at the *p* = 0.01 level. Furthermore, the distribution of these regions on the *Ovis aries* chromosome was analyzed by the karyoploteR package of Bioconductor (Gel and Serra, 2017). Functional annotation of CNVs was completed by ANNOVAR(Wang et al., 2010) and classified as intronic, exonic or intergenic.

### 2.3 Comparison with recent reports

To verify the reliability of our study, we compared the CNVR number and length of this study with three recently reports (Yuan et al., 2021; Huang et al., 2021; Salehian-Dehkordi et al., 2021). All these location information were annotated based on Oar_v4.0 reference genome.

### 2.4 Functional enrichment analysis of CNVR-harboring genes

CNVR-harboring genes were retrieved from the NCBI database by BioMart software (http://www.biomart.org/), and completely or partially (≥50%) overlapping genes were all reserved for later analysis. GO (Ashburner et al., 2000) and KEGG (Kanehisa and Goto, 2000) functional enrichment analyses were performed according to Huang et al. (Huang et al., 2009). The *p* value was calculated and subjected to FDR correction. The merged CNVRs were compared with QTLs in the animal QTL database (https://www.animalgenome.org/cgi-bin/QTLdb/OA/index), to further assess the CNVRs that were correlated with economic traits in three ecotypes of Tibetan sheep.

### 2.5 Sweep selective analysis of the CNVR

Tibetan sheep can be divided into three different ecotypes or populations according to their phenotypes and habitat environment. The three ecotypes have formed their own special characters under selection throughout long-term domestication and adaptation in the plateau. Therefore, the Vst (Redon et al., 2006) was calculated similar to population differentiation index Fst using the equation: Vst = (Vtotal–[Vpop1×Npop1+Vpop2×N pop2]/Ntotal)/Vtotal, where Vtotal is the total variance in LRRs (log-R ratios) of SNPs (within a defined CNVR) estimated among individuals of two populations, Vpop is the variance for each respective population, Npop is the sample size for each respective population, and Ntotal is the total sample size of the two population. Subsequently, the CNVRs with the top five Vst values were selected as candidate CNVRs, and functional enrichment analysis of these regions was performed.

### 2.6 qPCR and high depth resequencing validation

Quantitative real-time PCR was employed to validate the accuracy of CNVs detected in our study. 11 CNVR-harboring genes representing 5 deletion and 6 duplication types were selected. 9 sheep samples from the three TS populations were used for qPCR validation. The primers were designed using Primer 5 software based on our obtained genomic sequences, and DGAT1 gene was chosen as a reference gene (all primer information were shown in Supplementary Table S2 qPCR was performed using the TB Green PCR reagent kit (Takara Bio). Three replicates per sample and blank controls were required in the PCR. The 2^−ΔΔCt^ method was used to calculate the copy number of the targeted genes [38–40].

## 3 Results

### 3.1 The landscape of copy number variation in Tibetan sheep

The total number of raw reads obtained for a single sheep varied between 20,759,076,000 (a highland-type sheep) and 24,338,034,900 (a valley-type sheep), and the high-quality data reached 516.242 GB, with an average of 21,510,086,113 bp per individual. The average depth was 7.10× ± 0.30×, and the coverage rate was 83.72% ± 1.63% at least 4× (Supplementary Table S1).

**FIGURE 1:**
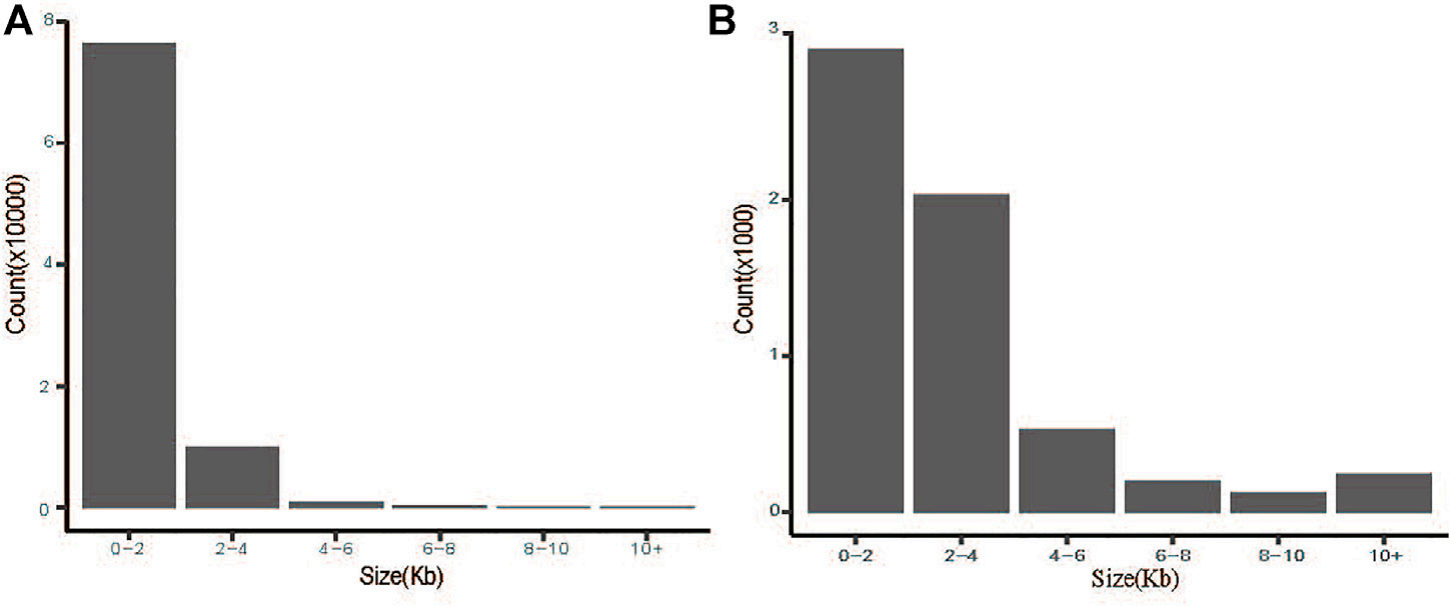
Size distribution of CNVs **(A)** and CNVRs **(B)** in Tibetan sheep.

The CNVs were detected using CNVcaller as described by Wang et al. with default parameters (Wang et al., 2017). A total of 87,832 CNVs were detected in 24 samples (Figure 1). The number of duplication and deletion events was 21,830 and 7479 for HTS, 21,588 and 7079 for OTS, and 21,816 and 8040 for VTS, respectively. The average lengths were 1.96, 1.95 and 1.99 kb (Table 1, Supplementary Table S3). The frequency of duplication events was approximately 3 times higher in all three populations than that of deletions. Moreover, as shown in Figure 1A, more than 86.9% of CNVs were distributed within 0–2 kb, 11.3% were within 2–4 kb, and less than 2% were greater than 4 kb in size.

**TABLE 1:** Summary of CNVs and CNVRs identified in three Tibetan sheep populations.

	Sheep	Sample	Count	Duplication	Deletion	Length (Mb)	Average (kb)	Percentage^a^ (%)
	Type	Number						
CNVs	HTS	8	29309	21830	7479	57.40	1.96	–
	OTS	8	28667	21588	7079	55.98	1.95	–
	VTS	8	29856	21816	8040	59.27	1.99	–
CNVRs	HTS	8	2096	1575	521	7.79	3.72	0.3
	OTS	8	2005	1457	548	7.65	3.82	0.3
	VTS	8	2003	1484	519	7.58	3.78	0.29

a Percentage of chromosome by CNVRs (%).

**FIGURE 2:**
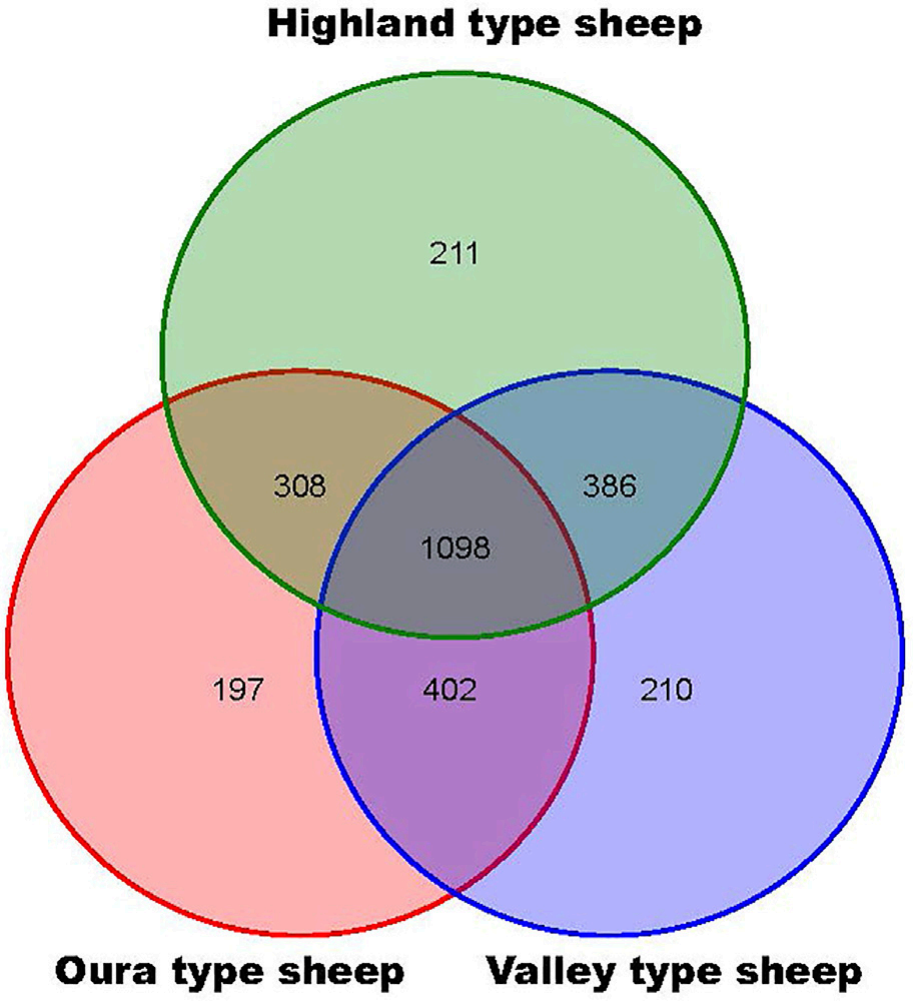
Venn diagram of CNVR numbers identified in three Tibetan sheep populations.

**FIGURE 3:**
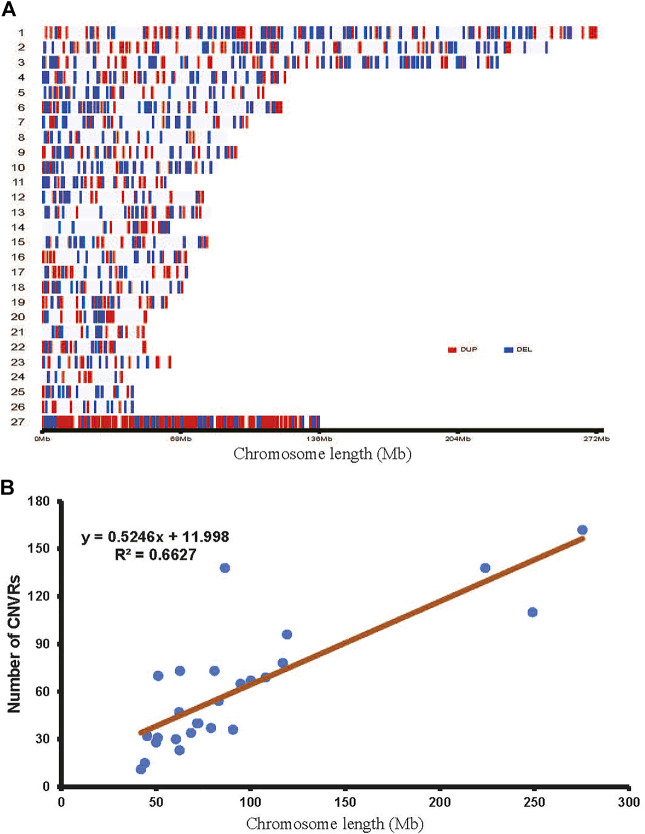
Genomic landscape of CNVRs in Tibetan sheep.

A total of 2777 CNV regions (CNVRs) were obtained by merging overlapping CNVs in the three populations, and the numbers of duplication types in HTS, OTS and VTS were 1575, 1457 and 1484, respectively. Meanwhile, the numbers of deletion types were 521, 548 and 519. The duplication to deletion ratio of CNVRs is consistent with that of the CNVs. The average size of these CNVRs in the three populations was more than 3 kb, accounting for 0.3% of the sheep genome (Oar_v4.0, Table 1). The size distribution of all the CNVRs showed an L-shaped pattern, with 58.2% of the CNVRs located within 1–2 kb, 28.7% within 2–4 kb, 6.5% within 6–8 kb, and others greater than 8 kb in size (Figure 1B). Moreover, as shown by the Venn diagram, 1002 CNVRs were shared by all three populations: approximately 203 CNVRs were distributed uniquely in HTS, 201 CNVRs were distributed in VTS, and 183 CNVRs were distributed in OTS (Figure 2, Supplementary Table S4). The results indicated that these CNVRs were nonuniformly distributed across each chromosome (Figure 3A), and the number of CNVRs had a significant positive linear relationship with the corresponding chromosome size (R2 = 0.66, Figure 3B). More CNVRs were distributed on OARX (1180), OAR1 (162), OAR 3 and OAR 10 (138), and the fewest CNVRs were distributed on OAR 24 (11), as shown in Supplementary Figure S1 and Supplementary Table S5. Functional classification showed that 1839 CNVRs were located in intergenic regions, 812 were contained within introns, and the other 85 were located in exonic regions.

### 3.2 Comparison with recent reports on CNVs in sheep

The results of our study were compared with three recently reported studies on sheep CNVRs. As shown in Table 2, the CNVR count detected in sheep varied notably from 1217 to 24534. Accordingly, the CNVR count overlapping with this study varied from 145 to 421. The average CNVR length was several kb using resequencing platform, but increased to hundreds kb based on BeadChip platform.

**TABLE 2:** Comparison of our study with three recent sheep CNV reports.

Study	Platform	Breed	Sample	CNVR count	Average CNVR length (Kb)	Total CNVR length (Mb)	Overlapping CNVR count with present study	Overlapping
								Percentage
Huang et al. (2021)	Resequencing	66	412	24534	3.58	87.92	421	1.72%
Yuan et al. (2021)	Illumina HiSeq 4000	4	32	7228	7.56	56.06	305	4.22%
Salehian-Dehkordi et al. (2021)	BeadChip 600K	67	2059	1217	201.31	245	145	11.91%
This Study	HiSeq X-Ten	3	24	2777	3.77			

### 3.3 Functional annotation of CNVRs

Functional enrichment analysis was performed for all 2777 detected CNVRs. Among the CNVRs shared by the three populations of Tibetan sheep, 56 GO terms (*p* < 0.01) were enriched, including 23 biological processes, 8 cellular components and 25 molecular functions (Supplementary Table S6). These GO terms involved ion transport (GO:0006811, GO:0006812), sensory perception system (GO:0007600, GO:0050906, and GO:0050907), gas transport (GO:0015669), and pigmentation (GO:0032400, GO:0051875). KEGG pathway analysis showed that the shared CNVR-harboring genes were enriched in 18 pathways (*p* < 0.05, Supplementary Table S7), including disease defense (ko04612, ko05332, ko05330, ko05320 and ko04672), nutrition metabolism (ko02010, ko00020, and ko00910), hematopoietic cell lineage (ko04640), and ABC transporters (ko02010), among others. In particular, the CNVR-harboring genes specifically distributed in HTS mainly participated in pigmentation (GO:0043473, GO:0033059, GO0048753, GO:0051875, and GO:0032400) processes.

### 3.4 QTLs overlapping with identified CNVRs

To further reveal the CNVRs associated with sheep economic traits and confirm their hereditary effects, the detected CNVRs were compared with QTLs in the sheep QTL database. We found 188 CNVRs overlapping with 97 quantitative trait loci (QTLs), including milk production and quality (61 CNVRs), fecal egg counts (56 CNVRs), tail fat deposition (21 CNVRs), immunoglobulin A level (20 CNVRs), bone development (14 CNVRs), growth and carcass traits (36 CNVRs) (Supplementary Table S8). Some CNVR-harboring genes related to slaughter performance were uncovered, such as PCDH15 (protocadherin related 15) gene located in body weight (slaughter) QTL (95871), carcass bone percentage QTL (95870), hot carcass weight QTL 95872) and muscle weight in carcass QTL (95853); APP (Amyloid precursor protein) gene located in muscle weight in carcass QTL 95851) and total muscle area QTL (95852). Meanwhile, the *ASIP*, LOC101111988 and LOC105606907 genes located in the tail fat deposition QTL (127012) were also detected.

### 3.5 CNVRs diverging among populations

**FIGURE 4:**
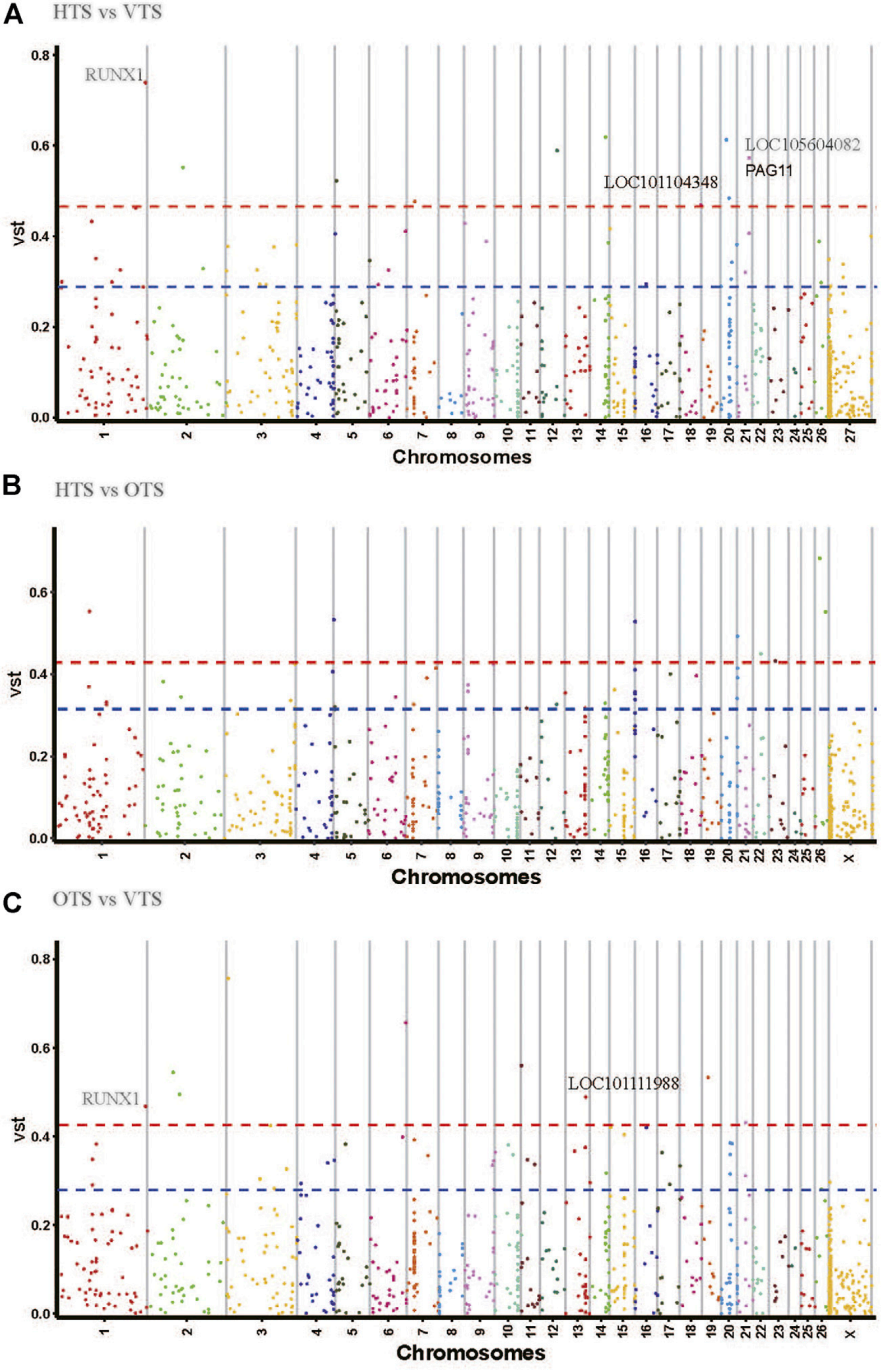
Genome wide Vst value plots for CNVRs in three population pairs.

The Vst statistic was used to analyze the population differentiation of CNVRs among HTS, VTS and OTS. This method is similar to Fst in estimating the population-specific selective pressure at the gene level but uses the protein-coding genes annotated by CNVR. The average values of Vst across all of the detected responding CNVRs were 0.1061 for ‘HTS vs. OTS’, 0.099 for ‘HTS vs. VTS’, and 0.096 for ‘VTS vs. OTS’ (Supplementary Table S9). As shown in Figure 4 and Supplementarty Table S9, the divergent CNVRs were distributed unevenly on the chromosome. Eighteen CNVR overlapping genes or loci, including RUNX family transcription factor 1 (*RUNX1*), glutamate receptor 4 (*GRIA4*), LOC101104348, LOC105604082, pregnancy-associated glycoproteins 11 (*PAG11*) and LOC106990378*,* were the top 1% of genes that showed significant divergence between HTS and VTS (Figure 4A). There were 15 overlapping genes or loci, including *RAPGEF1*, *MED27,* LOC105615522, *RUNX1*, LOC101113153, LOC105607734 and LOC101111988 (located upstream of *ASIP*), among others, which were the top 1% genes highly differentiated between the VTS and OTS (Figure 4C; Table 3). Among them, only *RUNX1*, LOC101104348, LOC105604082, *PAG11* and LOC101111988 were located in exonic or intronic regions. Unfortunately, few significantly divergent genes located in exonic or intronic regions were detected between HTS and OTS. Notably, *RUNX1* was detected in both the ‘HTS vs. VTS’ pairs and ‘VTS vs. OTS’ pairs. The CNV within *RUNX1* was duplication type with the length of 2400 bp and located in the intronic region (Table 3). It is worth noting that *ASIP*, playing a vital role in coat color, also showed significant variation between ‘VTS vs. OTS’ pairs and ‘HTS vs. OTS’ pairs.

**TABLE 3:** CNVR-harboring genes showing high divergence in two population pair s.

	Chr	Start	end	Type	Gene type	Vst	Symbol	Description
HTS vs. VTS	1	264846001	264848400	DUP	intron	0.739	*RUNX1*	runt-related transcription factor 1
	18	66356001	66358800	DUP	exon	0.4679	LOC101104348	uncharacterized LOC101104348
	21	37542001	37545600	DUP	exon	0.5726	LOC105604082	uncharacterized LOC105604082
	21	37542001	37545600	DUP	exon	0.5726	*PAG11*	pregnancy-associated glycoprotein 11
OTS vs. VTS	1	264846001	264848400	DUP	intron	0.468	*RUNX1*	runt-related transcription factor 1
	13	62932801	62934800	DUP	exon	0.4892	LOC101111988	agouti-signaling protein

### 3.6 CNV validation by qPCR

qPCR was used to verify the accuracy of CNVR predictions. 11 CNVRs including 5 deletions and 6 duplications were selected according to their Vst value (Supplementary Table S9). qPCR results showed that the five deletions (LOC101111526*, PLCB1*, *GUCY1A2*, *GRIA4*, and *TMEM144*) and six duplications (LOC105602432, *RUNX1*, *PAG11*, LOC101113153, and *ASIP*) were all consistent with the results based on whole genome sequencing prediction (Supplementary Figure S2).

## 4 Discussion

To date, there are many reports regarding the detection CNVs of sheep using aCGH, SNP array and genome resequencing (Fontanesi et al., 2011a; Jenkins et al., 2016; Di Gerlando et al., 2022). In the present study, we obtained 2777 CNVRs, including 1965 duplications and 812 deletions. The detected CNVR counts is higher than 619 reported by Yang et al. (Yang et al., 2018), comparable to 2394, 3488, and 4301 reported by Guo et al. (Guo et al., 2018), and Cheng et al. (Cheng et al., 2020), but far less than 24,534 claimed by Huang et al. (Huang et al., 2021). The CNVRs accounted for ∼0.3% of the sheep reference genome in our research. This coverage ratio is comparable with previous reports (Ma et al., 2015; Cheng et al., 2020) but lower than the 10% reported by Salehian-Dehkordi et al. (Salehian-Dehkordi et al., 2021). Consistent with the other reports, the number of CNVRs had a significant positive linear relationship with the corresponding chromosome size (Yuan et al., 2021). Meanwhile, the counts of overlapping CNVRs ranged from 145 to 421, which was comparable with Yuan et al. (Yuan et al., 2021). aCGH arrays and SNP chips were all constructed based on limited known probes. The limited probe resolution restricts the top ceiling of the CNV number that can be identified. Besides, they can’t be used to identify new CNVs especially for less studied native breed (Jenkins et al., 2016). Due to the difference in CNV detection platforms, the detected CNVs vary widely in different studies. In terms of the CNVs captured by aCGH and SNP chips, the number usually ranges from dozens to hundreds, and the average length is approximately 100–300 kb (Liu et al., 2013; Ma et al., 2015; Wu et al., 2015; Jenkins et al., 2016; Zhu et al., 2016). In addition to the detection platform and algorithm, the tested breed and individual size used were other considerable factors that results in the CNVRs counts obtained (Sudmant et al., 2015; Yuan et al., 2021; Huang et al., 2021; Prunier et al., 2022). In addition, more deletion evens than duplication were detected in this research, which is similar to Huang, et al. (Huang et al., 2021). But this result is contrary to Yuan et al. (Yuan et al., 2021), that’s may due to CNVcaller were more sensitive in identifying duplication (Wang et al., 2017). Moreover, it may be the characteristic of Tibetan sheep.

The GO enrichment analysis showed that the detected CNVRs harboring genes shared by the three populations were significantly enriched in ion transport, sensory perception system, gas transport and pigmentation. Among them, sensory perception was also significantly enriched in yaks (Qiu et al., 2012; Hui et al., 2019) and other sheep breeds (Ma et al., 2015; Yuan et al., 2021). And this was even observed in *Rangifer tarandus caribou*, a wild boreal ruminant (Prunier et al., 2022). The three TS populations live in alpine grasslands where the weather conditions are much harsher. As yak, TS faced the similar living conditions especially the lack of herbage in cold season. The well-developed sensory perception system was important to improve their ability to acquire food and avoid noxious weeds (Qiu et al., 2012). In the KEGG pathway analysis, it is noteworthy that the CNVR-harboring genes shared by the three populations were significantly enriched in nutrition metabolism, ABC transporters, disease defense and hematopoietic cell lineage. The enrichment of nutrient metabolism terms, including nitrogen metabolism, citrate cycle, and bile secretion, was important for the digestion of nutrients in TS, especially in the cold season. Similar results showing the enrichment of CNVR-harboring genes in ABC transporters were also reported in humans (Sun et al., 2014; Dron et al., 2018), cattle (Liu et al., 2011; Lee et al., 2013; Upadhyay et al., 2017) and goats (Guan et al., 2020). In mammals, ABC transporters can carry a broad array of endogenous metabolites, such as amino acids, peptides, and sugars, across lipid membranes, which facilitate the absorption and utilization of these nutrients. Overall, the number of enriched CNVR overlapping genes associated with forage intake and consumption may be helpful for their adaptation to the local environment (Allen et al., 2009).

Notably, the olfactory transduction pathway was significantly enriched specifically in OTSs. Enrichment of the olfactory transduction pathway has been reported in cattle Zhang et al., 2015; Gao et al., 2017; Lemos et al., 2018; Mielczarek et al., 2018), yaks (Guang-Xin et al., 2020), sheep (Jiasen et al., 2013; Liu et al., 2013; Yuan et al., 2021) and goats (Guan et al., 2020). It has been revealed to influence food consumption (Ma, 2008) and as a factor to assess feed efficiency and performance in crossbred beef cattle (Abo-Ismail et al., 2014) and residual feed intake in pigs (Do et al., 2014). Meanwhile, more CNVR harboring genes were enriched in amino acid and VFA metabolism pathways in the OTS. In regards to the much better meat performance of OTS than HTS and VTS (Zhao, 2010). More CNVR harboring genes enriched in the olfactory transduction pathway, together with protein and energy metabolism pathways, may explain the better production performance in OTS.

QTLs, which contain genetic variants affecting the economic traits of domestic animals, can be used to select candidate CNV-overlapping genes that affect phenotypes in sheep (Hu et al., 2022). After integrating CNVs into QTLs, we found 188 CNVRs overlapping with 97 sheep QTL regions in this study. Many CNVs overlapping genes, such as *PCDH15*, *APP* and *GRID2*, are located in growth and carcass QTL regions. The PCDH15 gene was identified to be associated with the concentration of the neurotransmitter glutamate (Glu) in cattle (Chen et al., 2020). Zheng et al. indicated that homozygous *APP* deficiency leads to a 15%–20% reduction in body weight (Hui et al., 1995). An et al. reported that adipocyte-specific and mitochondria-targeted *APP*-overexpressing mice display increased body mass (An et al., 2019). *GRID2* was also identified as being associated with body weight in rats (Keele et al., 2018). So, these identified CNV harboring genes provide candidate molecular associated markers for future sheep breeding.

Selective sweeping can reveal putative regions that undergo environmental and artificial selection during local adaptation and domestication. To screen the critical CNVR significantly divergent between different populations, the pairwise Vst value was estimated (Chen et al., 2020; Salehian-Dehkordi H, 2021; Yuan et al., 2021). Here, the five CNVR harboring genes *RUNX1*, LOC101104348, LOC105604082, *PAG11* and LOC101111988 showed significant pairwise differentiation among HTS, OTS and VTS. *RUNX*1 has been reported overlapping with a CNV in Chaka sheep (Cheng et al., 2020). It is well known that *RUNX1* plays a crucial role in hematopoiesis, leukemogenesis and neural development. Lin et al. reported that GWAS hit SNPs associated with colostrum albumin concentration were enriched in *RUNX1* in Chinese Holsteins, and these mutations might initiate the hyperactivation of inflammatory and innate immunity (Lin et al., 2020). In pigs, the GWAS hit SNP within *RUNX1* is associated with the mean corpuscular volume level (Lee et al., 2016). Moreover, HIF-1α facilitates *RUNX1* transcriptional activity under hypoxic conditions and triggers hematopoietic stem cell differentiation, which ultimately improves oxygen transport to peripheral tissues (Lee et al., 2017). As we know, Chaka sheep is a cultivated breed mainly with Tibetan sheep as crossbreeding ewes and adapted to a low oxygen environment for a long time (Zhao, 2010). Hence, we speculated that CNVR-harboring *RUNX1* has undergone strict selection pressure in Tibetan and Chaka sheep, possibly helpful for their adaptation to a low oxygen environment at high altitudes. PAG11 is a pregnancy-associated glycoprotein that is expressed in the trophoblast of the ruminant placenta and influences embryo growth and survival (Shorten et al., 2018). In contrast to VTS, HTS always faces shortage of forage and cold environments in their late pregnancy stage. We thought the divergence of CNVR-harboring *PAG11* between HTS and VTS may be related to their different habitat conditions, especially in the pregnancy stage. Notably, CNVR-harboring LOC101111988, located upstream of *ASIP*, was divergent between VTS vs. OTS (Vst = 0.489) and HTS vs. OTS (Vst = 0.284). The ASIP gene has been widely studied in mammals for its effect on animal coat color. Individuals with normal or duplication alleles of the ASIP gene are generally white or gray coat color, but individuals with normal or single deletion alleles in *ASIP* almost entirely have solid-black coat color in sheep (Norris and Whan, 2008; Fontanesi et al., 2011b; Han et al., 2015). In our study, the duplication of the LOC101111988 in VTS and HTS might account for their mainly white coat color. Meanwhile, the deletion in LOC101111988 might be the basis for the primary brown covering color, especially in the neck in OTS.

## 5 Conclusion

In this study, the whole genome characteristics were described in three ecotypic populations of Tibetan sheep. A total of 87,832 CNV events and 2777 CNVRs covering 0.3% of the sheep genome were captured. A few CNVR-harboring genes, such as *PCDH15*, *APP*, *RUNX1*, *PAG11,* and *ASIP*, were uncovered and may be associated with body weight, environmental adaptation, fertility, and coat color. Above all, our results provide a valuable genome-wide variation resource in Tibetan sheep for the elucidation of the genetic basis underlying the distinct phenotypic traits and local adaptation of Tibetan sheep.

## Data Availability

The datasets presented in this study can be found in online repositories. The names of the repository/repositories and accession number(s) can be found below: https://ngdc.cncb.ac.cn/, CRA005573.
