# Supplementary figures and images for: Genome-wide analysis of CNVs in three populations of Tibetan sheep using whole-genome resequencing
Source: Front Genet. 2022 Sep 7;13:971464. doi: 10.3389/fgene.2022.971464 (PMC9490000; doi:10.3389/fgene.2022.971464)

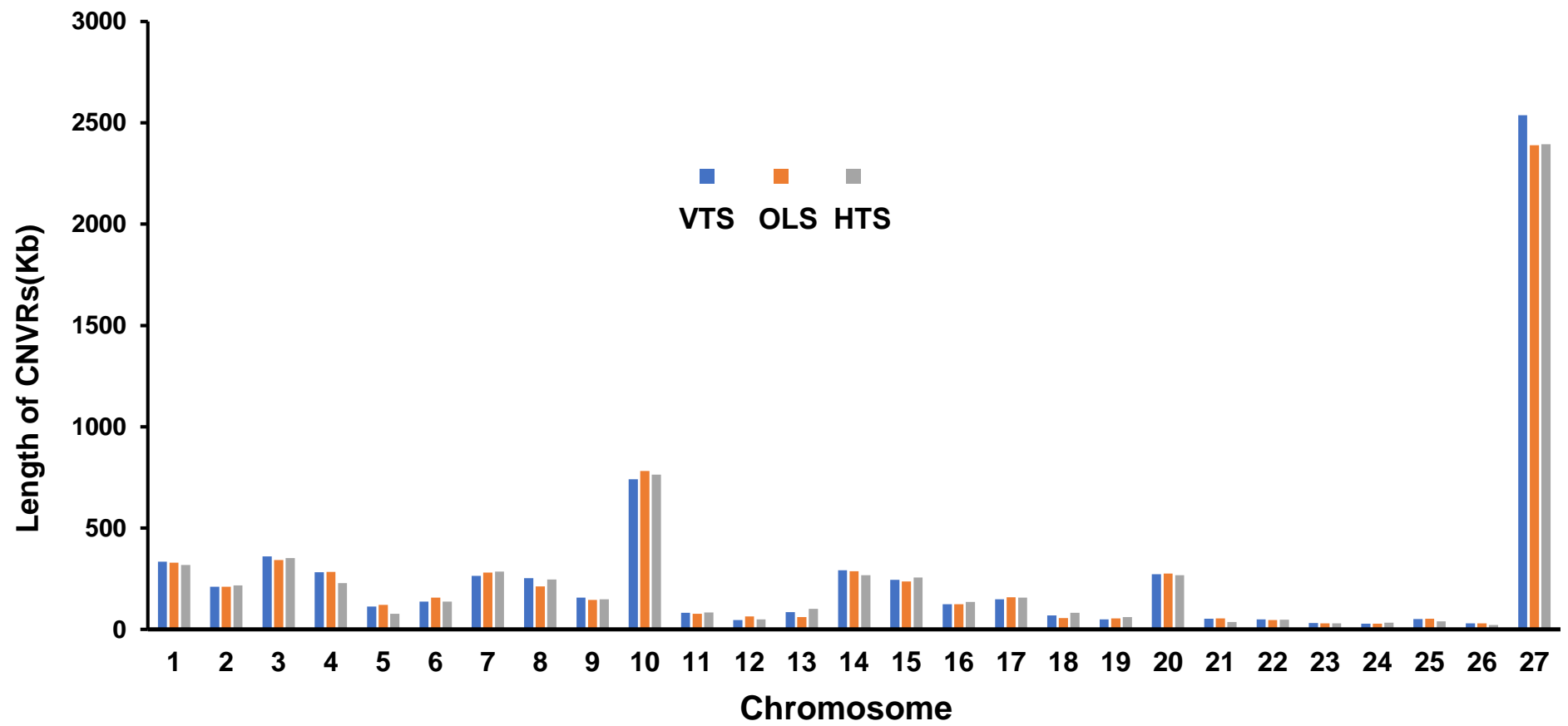

Supplement: Supplementary file 1 [file DataSheet1.ZIP › Additional Files/Figure S1. The length of CNVRs distributed on each chromosome in three TS populations.pdf]

Relative quantification Values

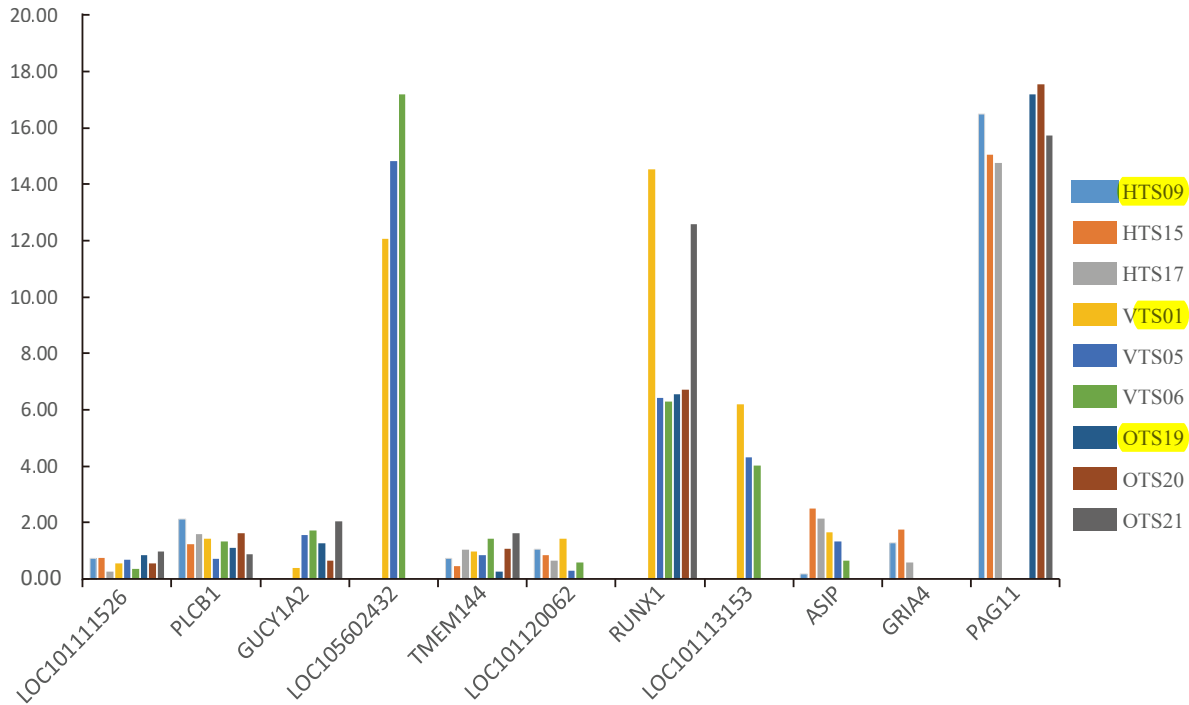

Supplement: Supplementary file 1 [file DataSheet1.ZIP › Additional Files/Figure S2. The qPCR validation results for 10 selected CNV loci.pdf]
